# P02.101. Vitamin D status of female healthcare employees of childbearing age

**DOI:** 10.1186/1472-6882-12-S1-P157

**Published:** 2012-06-12

**Authors:** G Plotnikoff, K Mullin, L Mahlke, S Calvin, M Finch, J Dusek

**Affiliations:** 1 Allina Center for Healthcare Innovation, Minneapolis, USA; 2 Allina Healthcare Employee Benefits , Minneapolis, USA; 3 Minnesota Perinatal Physicians, Minneapolis, USA; 4 University of Minnesota Carlson School of Business, Minneapolis, USA

## Purpose

To examine the relationship between vitamin D status, reported vitamin D intake and body mass index in female health care employees.

## Methods

Prospective observation study of 10,646 employees of a Midwestern integrated health care system who were measured for 25-OH-vitamin D by CLIA technology.

## Results

A total of 5,628 women (aged 15-49) met eligibility criteria. Of these, 1,710 (32.4%) did not meet 2010 ACOG or IOM vitamin D guidelines for vitamin D sufficiency (≥ 20 ng/ml); 3,684 (65.5%) did not meet 2010 international guidelines (≥ 30 ng/ml); and 4,874 (86.6%) did not meet 2011 Endocrine Society guidelines (40-60 ng/ml). Only 2,644 (46.97%) reported taking any vitamin D. For those participants who reported vitamin D3 intake equal to that found in prenatal and multivitamins (200-400 IUs) (n = 430), 17.7% had 25-OH-vitamin D levels <20 ng/ml, 59.5% had levels <30 ng/ml, and 85.3% had levels <40 ng/ml. Mean 25-OH-vitamin D serum levels and standard deviations for higher reported vitamin D3 daily intakes of 1,001-2,000 IUs, 2001-3,000 IUs and 3,001-4,000 IUs and >4,000 IUs were 34.09 ng/ml (12.79), 39.52 ng/ml (16.16), 38.57 ng/ml (17.06) and 37.98 ng/ml (16.40), respectively. For all of these reported intakes, women with a BMI ≥ 30 exhibited significantly lower 25-OH-vitamin D status compared to those women with BMI < 30 (p <.0001).

## Conclusion

Female healthcare workers of child bearing age demonstrate a high incidence of vitamin D deficiency. Daily prenatal or multivitamin supplementation does not ensure adequate 25-OH-vitamin D levels. A BMI ≥30 represents a substantially increased risk of suboptimal 25-OH-vitamin D status. Reported daily intake of >4,000 IUs did not result in elevated serum levels of vitamin D. These findings have substantial public health implications as vitamin D deficiency has been associated with increased obstetrical and perinatal risks including gestational diabetes mellitus, premature delivery and emergent c-section.

